# The Effect of p53 Status of Tumor Cells on Radiosensitivity of Irradiated Tumors With Carbon-Ion Beams Compared With γ-Rays or Reactor Neutron Beams

**DOI:** 10.14740/wjon941w

**Published:** 2015-08-27

**Authors:** Shin-ichiro Masunaga, Akiko Uzawa, Ryoichi Hirayama, Yoshitaka Matsumoto, Yoshinori Sakurai, Hiroki Tanaka, Keizo Tano, Yu Sanada, Minoru Suzuki, Akira Maruhashi, Koji Ono

**Affiliations:** a Particle Radiation Biology, Research Reactor Institute, Kyoto University, 2-1010, Asashiro-nishi, Kumatori-cho, Sennan-gun, Osaka 590-0494, Japan; b Research Center for Charged Particle Therapy, National Institute of Radiological Sciences, 4-9-1, Anagawa, Inage-ku, Chiba 263-8555, Japan; c Proton Medical Research Center, Faculty of Medicine, University of Tsukuba, 1-1-1, Tennodai, Tsukuba, Ibaraki 305-8575, Japan; d Radiation Medical Physics, Research Reactor Institute, Kyoto University, 2-1010, Asashiro-nishi, Kumatori-cho, Sennan-gun, Osaka 590-0494, Japan; e Particle Radiation Oncology, Research Reactor Institute, Kyoto University, 2-1010, Asashiro-nishi, Kumatori-cho, Sennan-gun, Osaka 590-0494, Japan

**Keywords:** p53 status, Quiescent cell, Carbon-ion beams, Reactor neutron beams, γ-rays

## Abstract

**Background:**

The aim of the study was to clarify the effect of p53 status of tumor cells on radiosensitivity of solid tumors following accelerated carbon-ion beam irradiation compared with γ-rays or reactor neutron beams, referring to the response of intratumor quiescent (Q) cells.

**Methods:**

Human head and neck squamous cell carcinoma cells transfected with mutant TP53 (SAS/mp53) or with neo vector (SAS/neo) were injected subcutaneously into hind legs of nude mice. Tumor-bearing mice received 5-bromo-2’-deoxyuridine (BrdU) continuously to label all intratumor proliferating (P) cells. They received γ-rays or accelerated carbon-ion beams at a high or reduced dose-rate. Other tumor-bearing mice received reactor thermal or epithermal neutrons at a reduced dose-rate. Immediately or 9 hours after the high dose-rate irradiation (HDRI), or immediately after the reduced dose-rate irradiation (RDRI), the tumor cells were isolated and incubated with a cytokinesis blocker, and the micronucleus (MN) frequency in cells without BrdU labeling (Q cells) was determined using immunofluorescence staining for BrdU.

**Results:**

The difference in radiosensitivity between the total (P + Q) and Q cells after γ-ray irradiation was markedly reduced with reactor neutron beams or carbon-ion beams, especially with a higher linear energy transfer (LET) value. Following γ-ray irradiation, SAS/neo tumor cells, especially intratumor Q cells, showed a marked reduction in sensitivity due to the recovery from radiation-induced damage, compared with the total or Q cells within SAS/mp53 tumors that showed little repair capacity. In both total and Q cells within both SAS/neo and SAS/mp53 tumors, carbon-ion beam irradiation, especially with a higher LET, showed little recovery capacity through leaving an interval between HDRI and the assay or decreasing the dose-rate. The recovery from radiation-induced damage after γ-ray irradiation was a p53-dependent event, but little recovery was found after carbon-ion beam irradiation. With RDRI, the radiosensitivity to reactor thermal and epithermal neutron beams was slightly higher than that to carbon-ion beams.

**Conclusion:**

For tumor control, including intratumor Q-cell control, accelerated carbon-ion beams, especially with a higher LET, and reactor thermal and epithermal neutron beams were very useful for suppressing the recovery from radiation-induced damage irrespective of p53 status of tumor cells.

## Introduction

It was shown that the p53 tumor suppressor gene serves a critical role in maintaining genomic stability during the cell cycle checkpoint in G1 and G2/M transition, and as an effector of DNA repair and apoptosis [1, 2]. Wild-type p53 is needed to activate apoptosis in sensitive cells in response to DNA damage [1, 2]. These actions of p53 are potentially critical in determining the effectiveness of ionizing radiation and/or chemotherapeutic agents. p53 is mutated in a majority of human solid tumors and plays a central role in the cellular response to DNA-damaging treatments like ionizing radiation, chemotherapy or hypoxic stress [3]. Hypoxic stress also induces p53 protein accumulation and p53-dependent apoptosis, but does not induce p53-dependent cell cycle arrest [3]. Loss of p53 function may result in resistance to DNA-damaging agents, including ionizing radiation and hypoxic stress [1, 3]. Actually, mutations in the p53 tumor suppressor gene have an impact on the clinical course of several human cancers: patients with cancers harboring p53 mutations often have a worse prognosis than those with tumors harboring wild-type p53 [1, 3]. Thus, the genetic and functional status of the p53 gene is an important factor in guiding therapeutic strategies for cancer patients.

Meanwhile, charged particle beams, including protons and heavy ions, can offer improved dose conformation to the target volume compared with photon or neutron radiotherapy, with better sparing of normal tissue structures close to the target [4]. In addition, ion beams heavier than helium exhibit a strong increase in linear energy transfer (LET) in the Bragg peak compared with the entrance region [4]. These physical properties are very advantageous in radiotherapy.

Also, high LET radiation, including neutrons, is more effective [5] than low LET X- or g-radiation at inducing biologic damage. High LET radiation results in a greater relative biologic effectiveness (RBE) value for cell killing, a reduced oxygen effect [6], and a reduced dependence on the cell cycle [7, 8], making it potentially superior to low LET radiation in the treatment of malignant tumors. The reactor thermal and epithermal neutron beams available at our institute have also been shown to have a significantly greater RBE value than γ-rays in irradiated tumor cells *in vivo* [9]. Owing to the selective physical dose distribution and enhanced biologic damage in target tumors, particle radiotherapy with protons or heavy ions has gained increasing interest worldwide, and many clinical centers are considering introducing radiotherapy with charged particles. However, almost all these biologic advantages of charged particle beams were determined only from the effects on tumor cell populations as a whole using *in vitro* cell cultures or *in vivo* solid tumors [4].

Many cells in solid tumors are quiescent (Q) *in situ* but are still clonogenic [10]. The Q tumor cell population has been thought to be more resistant to low LET radiation because of its much larger hypoxic fraction and greater potentially lethal damage repair (PLDR) capacity than the proliferating (P) tumor cell population, mainly determined by the characteristics of plateau-phase-cultured cells *in vitro* [10]. To date, using our method for selectively detecting the response of intratumor Q cell populations *in vivo*, we have already shown that almost all these characteristics can also be applied to Q state cells within solid tumors *in vivo* [11].

In this study, we examined the characteristics of radiosensitivity in the total (P + Q) and Q cell populations in solid tumors irradiated with 290 MeV/u accelerated carbon-ion beams at varying LET values in a 6-cm spread-out Bragg peak (SOBP) installed at the National Institute of Radiological Sciences (Chiba, Japan) compared with irradiation with ^60^C γ-rays and reactor thermal and epithermal neutron beams at our institute with our method for selectively detecting the response of Q cells within solid tumors [11], using two different tumor cell lines with identical genetic backgrounds except for p53 status.

## Materials and Methods

### Cells, tumors and mice

The human head and neck squamous cell carcinoma cell line SAS (JCRB, Tokyo) was cultured at 37 °C in Dulbecco’s modified Eagle’s medium (DMEM) containing 20 mM 2-[4-(2-hydroxyethyl)-1-piperazinyl]ethanesulfonic acid (HEPES) and 12.5% fetal bovine serum in a conventional humidified 5% CO_2_ incubator. SAS cells show the phenotype of wild-type p53 in radiation- and heat-induced signal transduction [12, 13]. Plasmid pC53-248, which contains an mp53 gene (codon 248, from Arg to Trp) producing a dominant negative mp53 protein, and plasmid pCMV-Neo-Bam, which contains a neo-resistance marker, were provided by B. Vogelstein (Johns Hopkins Oncology Center, Baltimore, MD). These plasmids were linearized with HindIII. Confluent SAS cells, approximately 2 × 10^6^ cells in a 75-cm^2^ flask, were trypsinized, and the resulting cell suspension in phosphate-buffered saline (PBS) (1 mL) was transferred into an electroporation chamber. Cells were supplemented with linearized DNA (10 μg/10 μL of pC53-248 or pCMV-Neo-Bam), and electroporated three times at 600 V. After standing for 30 min at room temperature, cells were plated onto dishes 10 cm in diameter in DMEM and incubated at 37 °C. Forty-eight hours later, cells were treated with G418 (geneticin, 200 μg/mL, Sigma Chemical Co., St. Louis, MO), an agent for selection of transfected clones, and then incubated at 37 °C for 14 days to allow colony formation. Colonies resistant to G418 were isolated with cloning cylinders. Through these manipulations, two stable transfectants SAS/mp53 and SAS/neo were established. SAS/neo cells have a functionally wild-type p53 protein, and SAS/ mp53 cells express a dominant-negative p53 protein. The procedure used for transfection is described in detail elsewhere [12, 13].

Cells were collected from exponentially growing cultures, and approximately 5.0 × 10^5^ cells were inoculated subcutaneously into both hind legs of 6- to 7-week-old syngeneic female Balb/cA nude mice. Three weeks after inoculation, a tumor with a diameter of approximately 7 mm could be observed at each implanted site, whichever stable transfectant was used.

Meanwhile, in locally advanced or recurrent head and neck tumors, especially which are refractory to conventional cancer therapy including radiation therapy using low LET radiation X-rays, p53 status of the tumor cells is often mutated and the tumors often show hypoxic tendency rather than fresh and non-treated virgin tumors [14, 15]. In the treatment of inoperable refractory advanced and recurrent head and neck tumors, neutron capture therapy (NCT) has been performed since early 2001 as a clinical study at our research reactor institute [16, 17]. Since October 2012, NCT for inoperable refractory locally advanced and recurrent head and neck tumors has been conducted as a clinical trial.

### Labeling with 5-bromo-2’-deoxyuridine (BrdU)

Two weeks after tumor cell inoculation, mini-osmotic pumps (Durect Corporation, Cupertino, CA) containing BrdU dissolved in physiological saline (250 mg/mL) were implanted subcutaneously to label all P cells for 7 days. Administration of BrdU did not change the tumor growth rate. The tumors were approximately 7 mm in diameter on treatment. The labeling index (LI) after continuous labeling with BrdU was 48.4% (41.7-55.1%) (mean (95% confidence limit)) and 43.2% (37.0-49.4%) for SAS/neo and SAS/mp53 tumor cells, respectively, and reached a plateau level at these stages. Therefore, in this study, we regarded tumor cells not incorporating BrdU after continuous labeling as Q cells.

### Irradiation

After labeling with BrdU, the tumor-bearing mice underwent accelerated carbon-ion beam irradiation, γ-ray irradiation, or reactor thermal or epithermal neutron beam irradiation. The irradiation was performed with the tumor-bearing mice held in a specially designed device made of acrylic resin with the tail or both arms and legs firmly fixed with an adhesive tape, without anesthesia.

Carbon-12 ions were accelerated up to 290 MeV/u by the synchrotron of the Heavy Ion Medical Accelerator installed at the National Institute of Radiological Sciences, Chiba, Japan. The accelerator was originally set up mainly for radiotherapy for malignant solid tumors refractory to conventional cancer therapy [18]. The radiation dose-rate was regulated through the beam attenuation system, and irradiation was conducted using horizontal carbon beams with a dose rate of 1.0 Gy/min and 0.037 Gy/min for high dose-rate irradiation (HDRI) and reduced dose-rate irradiation (RDRI), respectively. The LET of the 290 MeV/u carbon beam with the 6-cm SOBP ranged from 14 keV/mm to > 200 keV/mm, depending on the depth. A desired LET beam was obtained by selecting the depth along the beam path using a Lucite range shifter. Carbon beams with 18 and 50 keV/mm LET were obtained at the middle of the plateau and at the middle of the SOBP. A desired irradiation field was obtained by the simultaneous use of an iron collimator and a brass collimator [19].

γ-Ray irradiation was performed with a ^60^Co γ-ray irradiator available at our institute at a dose-rate of 2.5 Gy/min, such as conventionally used for HDRI. RDRI was performed at a dose-rate of 0.039 Gy/min by maintaining an appropriate distance between the ^60^Co radiation source and the irradiated tumor-bearing mouse fixed within the specially constructed device.

Thermal and epithermal neutron irradiations were performed using a reactor neutron beam from our reactor with a cadmium ratio of 160 and 1.0, respectively. The neutron fluence was measured from the radioactivation of gold foil at the front and back of the irradiated tumors. Because the tumors were small and located just beneath the surface, the neutron fluence was assumed to decrease linearly from the front to the back of the tumors. Thus, we used the average neutron fluence determined from the values measured at the front and back. Contaminating γ-ray, including secondary γ-ray, doses were measured with a thermoluminescence dosimeter powder at the back of the tumors. For the estimation of neutron energy spectra, eight kinds of activation foils and 14 kinds of nuclear reactions were used. The absorbed dose was calculated using the flux/dose conversion factor [20]. The tumors were assumed to contain, in weight percentages, 10.7%, 12.1%, 2%, 71.4%, and 3.8% of hydrogen, carbon, nitrogen, oxygen, and other chemical elements, respectively [20].

The tumor-bearing mice fixed within the specially constructed devices were irradiated at a maximum neutron flux just in front of the neutron-radiating bismuth layer of the heavy water facility at our reactor. The neutron flux and Kerma rate of the thermal neutron beams were 2.0 × 10^9^ n/cm^2^/s and 98.5 cGy/h for the thermal neutron range (< 0.6 eV), 1.7 × 10^7^ n/cm^2^/s and 0.38 cGy/h for the epithermal neutron range (0.6 - 10 keV), and 3.4 × 10^6^ n/cm^2^/s and 22.9 cGy/h for the fast neutron range (> 10 keV), respectively. The contaminating γ-ray dose-rate was 100 cGy/h. All together, the irradiation dose-rate was 0.037 Gy/min. The corresponding values for the epithermal beams were 3.0 × 10^7^ n/cm^2^/s and 0.24 cGy/h for the thermal neutron range, 7.3 × 10^8^ n/cm^2^/s and 22.4 cGy/h for the epithermal neutron range, and 4.7 × 10^7^ n/cm^2^/s and 163 cGy/h for the fast neutron range. The contaminating γ-ray dose-rate was 62.0 cGy/h. As a whole, the irradiation dose-rate was 0.041 Gy/min [20]. Taking the values of the dose-rate used for accelerated carbon-ion beam and γ-ray irradiation into account, each neutron beam irradiation was thought to be equivalent to the RDRI. However, each dose-rate at each reactor neutron beam irradiation was the maximal value available at our reactor [20].

According to the International Commission on Radiation Units and Measurements Report 58 concerning dosimetry in intracavitary brachytherapy for uterine cancer, high, middle, and low dose-rate irradiation is defined as > 0.2 Gy/min (12 Gy/h), 0.033 - 0.2 Gy/min (2 - 12 Gy/h), and < 0.033 Gy/min (2 Gy/h), respectively [21]. Thus, the HDRI and RDRI used in the present study fit with the high dose-rate and middle dose-rate irradiation, respectively.

Each irradiation group also included mice that had not been pretreated with BrdU. The tumors were then excised immediately or 12 h after irradiation.

### Immunofluorescence staining of BrdU-labeled cells and observation of micronucleus (MN) formation

The tumors were excised from mice given BrdU, minced, and trypsinized (0.05% trypsin and 0.02% ethylenediamine-tetraacetic acid (EDTA) in PBS at 37 °C for 15 min). The tumor cell suspensions were incubated for 48 h in tissue culture dishes containing complete medium and 1.0 μg/mL of cytochalasin-B to inhibit cytokinesis while allowing nuclear division, and the cultures were then trypsinized and cell suspensions were fixed. After the centrifugation of fixed cell suspensions, the cell pellet was resuspended with cold Carnoy’s fixative. The suspension was then placed on a glass microscope slide and the sample was dried at room temperature. The slides were treated with 2 M hydrochloric acid for 45 min at room temperature to dissociate the histones and partially denature the DNA. The slides were then immersed in borax-borate buffer (pH 8.5) to neutralize the acid. BrdU-labeled tumor cells were detected by indirect immunofluorescence staining using monoclonal anti-BrdU antibody (Becton Dickinson, San Jose, CA) and fluorescein isothiocyanate (FITC)-conjugated antimouse immunoglobulin G (whole molecule) antibody (Sigma, St. Louis, MO). To observe double staining of tumor cells with green-emitting FITC and red-emitting propidium iodide (PI), cells on the slides were treated with PI and monitored under a fluorescence microscope.

The MN frequency in BrdU-unlabeled cells (Q cells at irradiation) could be examined by counting the micronuclei in the binuclear cells that showed only red fluorescence. The MN frequency was defined as the ratio of the number of micronuclei in the binuclear cells to the total number of binuclear cells observed [9, 11].

The ratios obtained in tumors not pretreated with BrdU indicated the MN frequency at all phases in the total (P + Q) tumor cell populations. More than 300 tumor cells and binuclear cells were counted to determine the apoptosis frequency and the MN frequency, respectively.

Needless to say, the induction of an MN requires division of the cell nucleus [22]. The duration of incubation with cytochalasin-B allowed Q cells to be recruited into the cell cycle. Thus, the optimal incubation period was determined so that the maximum rate of binuclear tumor cells could be observed. The frequencies of MN for BrdU-labeled cells were modified because the radiosensitization effect of the incorporated BrdU could potentially influence the frequencies in BrdU-labeled cells. Thus, the correct frequencies of BrdU-labeled cells without the BrdU effect are not able to be determined. During continuous labeling with BrdU, the shift of cells from P to Q population could result in labeled Q cells. These cells were excluded when we scored micronuclei in binuclear cells in tumor cells showing only red fluorescence by PI for DNA staining, because these cells were stained with FITC.

### Cell survival assay

The cell survival assay was also performed in mice given no BrdU using an *in vivo-in vitro* assay method. Tumors were disaggregated by stirring for 20 min at 37 °C in PBS containing 0.05% trypsin and 0.02% EDTA. The cell yield was 1.5 (1.2 - 1.8) × 10^7^/g and 3.4 (2.6 - 4.2) × 10^6^/g for SAS/neo and SAS/mp53 tumors, respectively.

To confirm the stability of transfectants SAS/neo and SAS/mp53, part of the tumor cell suspensions obtained after irradiation and tumor cells from part of the colonies grown through the *in vivo-in vitro* assay method were subjected to western blotting analysis for p53 and Bax proteins as described by Ota et al [23]. Not only the level, but also the function of p53 protein could be detected because the bax gene is a target of the p53 gene. As a result, it was confirmed that the p53 status of each transfectant was not changed by these experimental procedures. Three mice were used to assess each set of conditions and each experiment was repeated three times. To examine the differences between pairs of values, Student’s *t*-test was used when variances of the two groups could be assumed to be equal, otherwise the Welch *t*-test was used. P values were from two-sided tests.

## Results

Table 1 shows the plating efficiencies for the total tumor cell population and the MN frequencies without radiation for the total and Q cell populations in each tumor. Overall, SAS/mp53 tumor cells showed significantly lower plating efficiency in the total cell populations and significantly higher MN frequencies in both the total and Q cell populations (P < 0.05) than SAS/neo tumor cells. Further, Q cells showed significantly higher MN frequencies than the total cell population under each set of conditions in each tumor (P < 0.05).

**Table 1:** Plating Efficiency and Micronucleus Frequency at 0 Gy

	Total tumor cells	Quiescent cells
SAS/neo		
Plating efficiency (%)	45.5 ± 8.9^a^	
Micronucleus frequency	0.038 ± 0.006	0.056 ± 0.007
SAS/mp53		
Plating efficiency (%)	23.5 ± 4.1	
Micronucleus frequency	0.072 ± 0.008	0.111 ± 0.010

^a^Mean ± standard error (n = 6).

**Figure 1:**
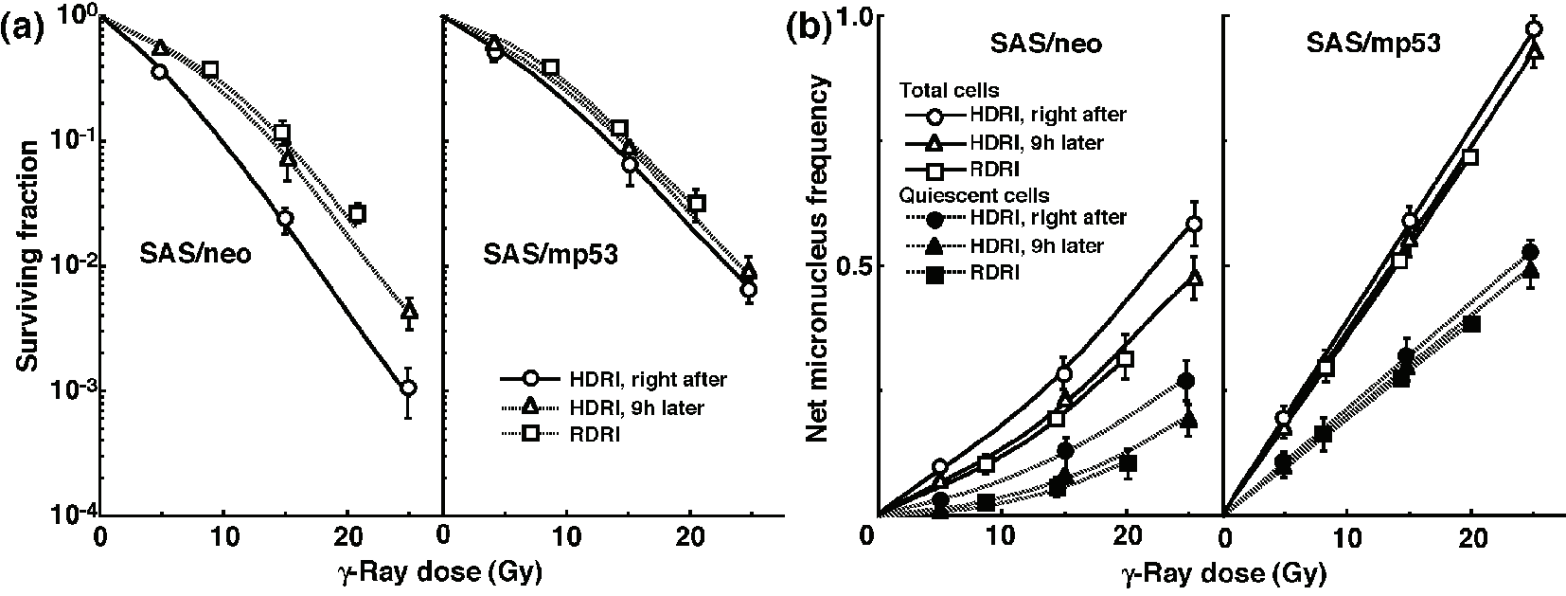
Surviving fractions and net micronucleus frequencies following γ-ray irradiation. The clonogenic cell survival curves for total tumor cell populations and the net micronucleus frequencies for total and quiescent cell populations immediately and 9 h after γ-ray irradiation with high dose-rate irradiation (HDRI) and immediately after γ-ray irradiation with reduced dose-rate irradiation (RDRI) are shown in (a) and (b), respectively. The left and right panels show SAS/neo and SAS/mp53 tumor cells, respectively. Bars represent standard errors (n = 6).

The clonogenic cell survival curves for total tumor cell populations and the net MN frequencies of total and Q cell populations after γ-ray irradiation with HDRI or RDRI are shown in Figure 1a and b, respectively. For baseline correction, we used the net MN frequency to exclude the MN frequency in non-irradiated control tumors. The net MN frequency was the MN frequency in the irradiated tumors minus that in the non-irradiated tumors. On the whole, SAS/mp53 tumor cells and Q tumor cells were more radioresistant than SAS/neo tumor cells and the total tumor cell population, respectively. The increase in the surviving fraction (SF) with the 9 h delayed assay, that is, PLDR, and the increase in the SF after RDRI were observed more clearly in SAS/neo than in SAS/mp53. The decrease in the net MN frequency with the 9 h delayed assay and the decrease in the net MN frequency after RDRI were more obvious in SAS/neo and Q cells than in SAS/mp53 and the total cell population, respectively.

**Figure 2:**
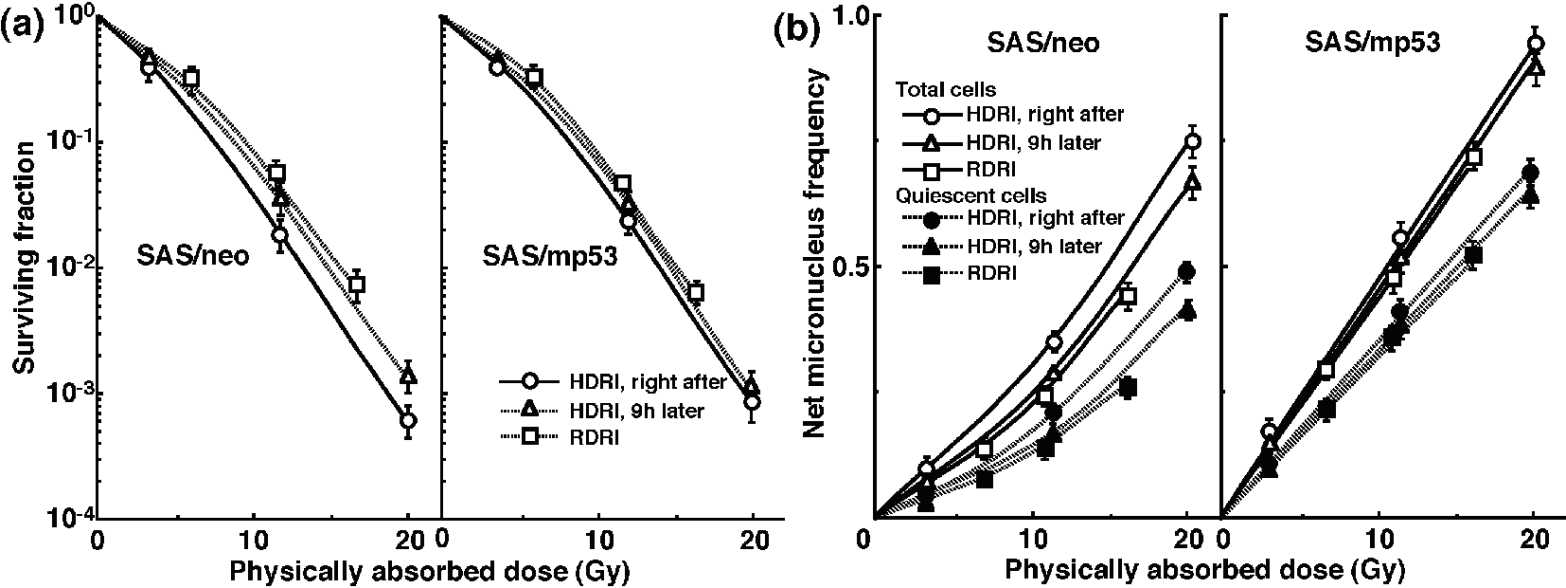
Surviving fractions and net micronucleus frequencies following accelerated carbon-ion beam (18 MeV/μm) irradiation. The clonogenic cell survival curves for total tumor cell populations and the net micronucleus frequencies for total and quiescent cell populations immediately and 9 h after irradiation using accelerated carbon-ion beams with a linear energy transfer of 18 MeV/μm with high dose-rate irradiation (HDRI) and immediately after irradiation with reduced dose-rate irradiation (RDRI) are shown in (a) and (b), respectively. The left and right panels show SAS/neo and SAS/mp53 tumor cells, respectively. Bars represent standard errors (n = 6).

**Figure 3:**
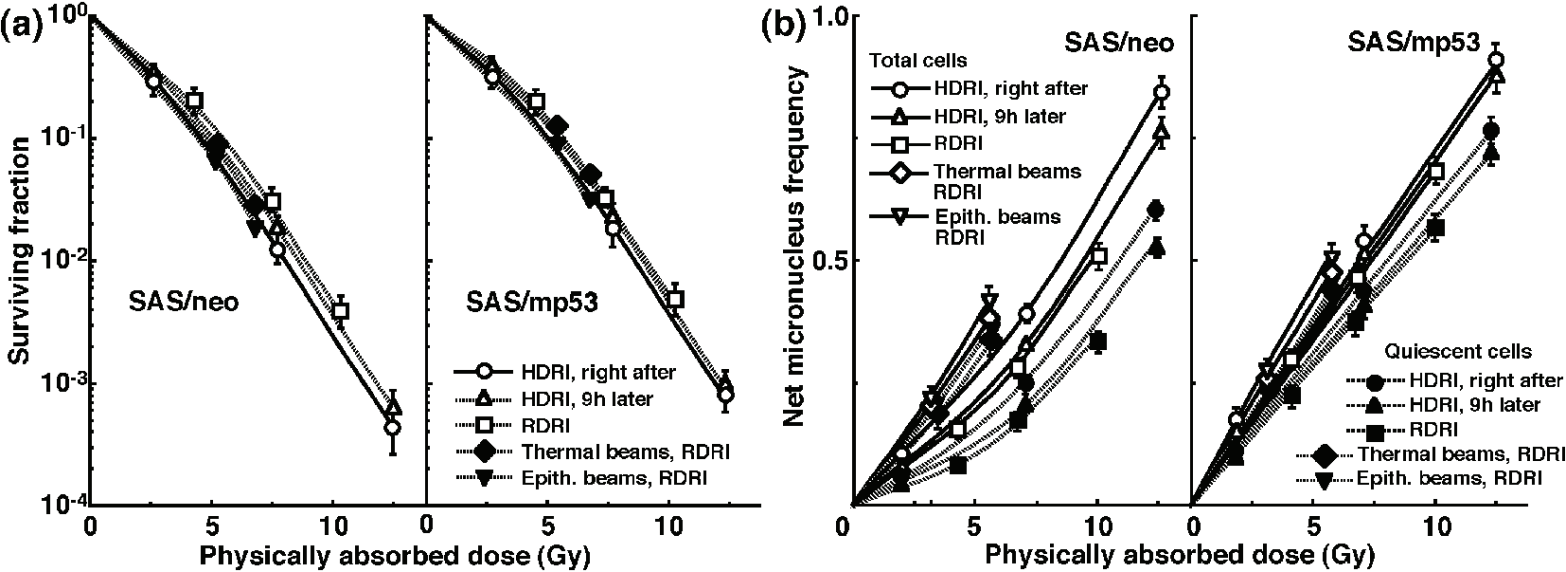
Surviving fractions and net micronucleus frequencies following accelerated carbon-ion beam (50 MeV/μm) irradiation. The clonogenic cell survival curves for total tumor cell populations and the net micronucleus frequencies for total and quiescent cell populations immediately and 9 h after irradiation using accelerated carbon-ion beams with a linear energy transfer of 50 MeV/μm with high dose-rate irradiation (HDRI) and immediately after irradiation with reduced dose-rate irradiation (RDRI) are shown in (a) and (b), respectively. In addition, the cell survival curves for total cells and the net micronucleus frequencies for total and quiescent cells immediately after irradiation using reactor thermal and epithermal neutron beams are also shown in (a) and (b), respectively. The left and right panels show SAS/neo and SAS/mp53 tumor cells, respectively. Bars represent standard errors (n = 6).

The clonogenic cell survival curves for total tumor cell populations and the net MN frequencies of total and Q cell populations following HDRI or RDRI using carbon-ion beams with 18 keV/μm LET are shown in Figure 2a and b, respectively. The clonogenic cell survival curves for total tumor cell populations and the net MN frequencies of total and Q cell populations following HDRI or RDRI using carbon-ion beams with 50 keV/μm LET are also shown in Figure 3a and b, respectively, including the data for immediately after RDRI with reactor thermal or epithermal neutron beams. On the whole, in terms of the SF, the difference in sensitivity between SAS/neo and SAS/mp53 tumors was reduced through employing carbon-ion beams instead of γ-rays. The difference in sensitivity between Q and total tumor populations was also reduced through employing carbon-ion beams instead of γ-rays in terms of the MN frequency. The increase in the SF with the 9 h delayed assay and after RDRI were inhibited through using carbon-ion beam irradiation. The decrease in the net MN frequency with the 9 h delayed assay and after RDRI were also repressed with carbon-ion beam irradiation.

To evaluate the recovery capacity from the damage induced by HDRI or during RDRI in total and Q cell populations within these two tumors, dose modifying factors were calculated in both cell populations at various endpoints using the data given in Figures 1-3 (Table 2). Overall, regardless of the cell populations, SAS/mp53 tumor cells showed little recovery capacity under any irradiation conditions. In SAS/neo tumor cells, whether 9 h after HDRI or after RDRI, the recovery capacities following γ-ray irradiation were significantly greater in Q cell populations than total cell populations (P < 0.05). Carbon-ion beam irradiation inhibited the recovery from the damage induced by HDRI and through RDRI with a more remarkable inhibition in Q cells than in the total cell population, especially when carbon-ion beams with a higher LET were employed.

**Table 2:** Dose-Modifying Factors Due to a Delayed Assay or Reduced Irradiation Dose-Rate^a^

Employed Radiation beams	High dose-rate 9 h after	Reduced dose-rate
SAS/neo		
Surviving fraction = 0.03		
Total cells		
γ-rays	1.3 ± 0.1^b^	1.4 ± 0.1
Carbon beams (18 keV/μm)	1.2 ± 0.1	1.15 ± 0.1
Carbon beams (50 keV/μm)	1.15 ± 0.1	1.1 ± 0.1
Net micronucleus frequency = 0.1		
Total cells		
γ-rays	1.35 ± 0.1	1.45 ± 0.1
Carbon beams (18 keV/μm)	1.15 ± 0.1	1.2 ± 0.1
Carbon beams (50 keV/μm)	1.15 ± 0.1	1.15 ± 0.1
Quiescent cells		
γ-rays	1.45 ± 0.15	1.55 ± 0.15
Carbon beams (18 keV/μm)	1.3 ± 0.1	1.35 ± 0.1
Carbon beams (50 keV/μm)	1.25 ± 0.15	1.3 ± 0.1
SAS/mp53		
Surviving fraction = 0.03		
Total cells		
γ-rays	1.05 ± 0.1^b^	1.1 ± 0.1
Carbon beams (18 keV/μm)	1.05 ± 0.1	1.05 ± 0.1
Carbon beams (50 keV/μm)	1.05 ± 0.1	1.05 ± 0.1
Net micronucleus frequency = 0.1		
Total cells		
γ-rays	1.05 ± 0.1	1.1 ± 0.1
Carbon beams (18 keV/μm)	1.05 ± 0.1	1.1 ± 0.1
Carbon beams (50 keV/μm)	1.05 ± 0.1	1.05 ± 0.1
Quiescent cells		
γ-rays	1.05 ± 0.1	1.1 ± 0.1
Carbon beams (18 keV/μm)	1.05 ± 0.1	1.1 ± 0.1
Carbon beams (50 keV/μm)	1.05 ± 0.1	1.1 ± 0.1

^a^The ratio of the dose of radiation necessary to obtain each endpoint with a delayed assay or reduced dose-rate irradiation to that needed to obtain each endpoint with an assay immediately after high dose-rate irradiation. ^b^Mean ± standard error (n = 6).

To compare the cell survival curve between these two tumor cells, we calculated the dose ratios for SAS/mp53 tumor cells relative to SAS/neo tumor cells (Table 3). The factors were calculated by comparing the radiation doses to obtain SF = 0.03 in SAS/mp53 tumor cells with the doses required in SAS/neo tumor cells. Following γ-ray irradiation, the values were decreased by the delayed assay after HDRI or a decreasing irradiation dose-rate because of the apparent recovery from radiation-induced damage in SAS/neo tumor cells, compared with SAS/mp53 tumor cells that showed little recovery capacity. However, following carbon-ion or reactor thermal or epithermal neutron beam irradiation, the values were kept almost constant mainly because of efficiently suppressing recovery from radiation-induced damage in SAS/neo tumor cells.

**Table 3:** Dose-Modifying Factors for SAS/mp53 Relative to SAS/neo Tumor Cells^a^

High dose-rate immediately after	High dose-rate 9 h after	Reduced dose-rate
Surviving fraction = 0.03		
Total cells		
γ-rays (1.35 ± 0.1^b^)	1.15± 0.1	1.1 ± 0.1
Carbon beams (18 keV/μm) (1.35 ± 0.1^b^)	1.1 ± 0.1	1.05 ± 0.1
Carbon beams (50 keV/μm) (1.1 ± 0.1)	1.05 ± 0.1	1.0 ± 0.1
Thermal beams		1.1 ± 0.1
Epithermal beams		1.05 ± 0.1

^a^The ratio of the physical radiation dose of external beams necessary to obtain each endpoint in SAS/mp53 tumor cells to that needed to obtain each endpoint in SAS/neo tumor cells. ^b^Mean ± standard error (n = 6).

To evaluate the RBE of the carbon-ion beams with various LET values and the reactor thermal and epithermal neutron beams in the total and Q cell populations compared with γ-rays, we used the data given in Figures 1-3 (Table 4). Overall, for all irradiation conditions, the values of RBE for Q cells were significantly larger than those for the total cell population (P < 0.05). For carbon-ion beams, in the total and Q cell populations, the RBE values for immediately after RDRI were significantly larger than those for immediately after HDRI (P < 0.05). Those for 9 h after HDRI were also larger than those for immediately after HDRI, although not as large as those for immediately after RDRI. As the LET value increased (i.e., as the irradiated point became deeper within the SOBP of the carbon-ion beam), the values of RBE in the total and Q cells increased. The RBE values for the reactor thermal neutron beams were further higher than those for the carbon-ion beams with the higher LET value (50 keV/μm), and those for epithermal beams were greater than those for thermal beams.

**Table 4:** Relative Biological Effectiveness for Carbon Beams Compared With γ-Rays^a^ in Total and Quiescent Tumor Cells

LET values of carbon beams	High dose-rate immediately after	High dose-rate 9 h after	Reduced dose-rate
SAS/neo			
Surviving fraction = 0.03			
Total cells			
18 keV/μm	1.35 ± 0.1^b^	1.5 ± 0.15	1.5 ± 0.15
50 keV/μm	2.2 ± 0.2	2.6 ± 0.25	2.6 ± 0.25
Thermal			3.2 ± 0.3
Epithermal			3.7 ± 0.35
Net micronucleus frequency = 0.1			
Total cells			
18 keV/μm	1.6 ± 0.15	1.7 ± 0.15	1.7 ± 0.15
50 keV/μm	1.85 ± 0.2	2.45 ± 0.25	2.6 ± 0.25
Thermal			4.2 ± 0.4
Epithermal			4.7 ± 0.45
Quiescent cells			
18 keV/μm	1.9 ± 0.2	2.4 ± 0.25	2.4 ± 0.25
50 keV/μm	3.45 ± 0.35	4.2 ± 0.4	5.0 ± 0.5
Thermal			6.4 ± 0.65
Epithermal			7.3 ± 0.75
SAS/mp53			
Surviving fraction = 0.03			
Total cells			
18 keV/μm	1.6 ± 0.15^b^	1.6 ± 0.15	1.65 ± 0.15
50 keV/μm	2.65 ± 0.25	2.65 ± 0.25	2.7 ± 0.25
Thermal			3.1 ± 0.3
Epithermal			3.3 ± 0.35
Net micronucleus frequency = 0.1			
Total cells			
18 keV/μm	1.25 ± 0.1	1.2 ± 0.1	1.2 ± 0.1
50 keV/μm	1.3 ± 0.15	1.35 ± 0.15	1.4 ± 0.15
Thermal			2.3 ± 0.25
Epithermal			2.45 ± 0.25
Quiescent cells			
18 keV/μm	2.05 ± 0.2	2.4 ± 0.25	2.4 ± 0.25
50 keV/μm	1.6 ± 0.15	1.75 ± 0.15	1.85 ± 0.2
Thermal			3.8 ± 0.4
Epithermal			4.2 ± 0.4

^a^Ratio of radiation dose necessary to obtain each endpoint with γ-rays and radiation dose necessary to obtain each endpoint with carbon-ion beams. ^b^Mean ± standard error (n = 6).

Table 5 shows the dose ratios of Q cells relative to total tumor cell populations; these factors were used to compare the radiation doses necessary to obtain the net MN frequency of 0.1 in Q cells with the doses required in the total tumor cell populations. All the values of the dose ratios were significantly larger than 1.0 (P < 0.05). Carbon-ion beam irradiation especially with a higher LET efficiently reduced the difference in γ-ray sensitivity between the total and Q tumor cell populations, especially in SAS/neo tumors, due to a greater recovery capacity in Q cells than in the total cell population at 9 h after HDRI and after RDRI. Reactor thermal and epithermal neutron beam irradiation also efficiently reduced the difference in γ-ray sensitivity between the total and Q tumor cell populations following RDRI.

**Table 5:** Dose-Modifying Factors for Quiescent Relative to Total Tumor Cells^a^ at Net Micronucleus Frequency of 0.1

High dose-rate immediately after	High dose-rate 9 h after	Reduced dose-rate
SAS/neo		
γ-rays (2.3 ± 0.25^b^)	2.35 ± 0.25	2.4 ± 0.25
Carbon beams (18 keV/μm) (2.0 ± 0.2)	1.7 ± 0.15	1.65 ± 0.15
Carbon beams (50 keV/μm) (1.9 ± 0.1)	1.75 ± 0.15	1.9 ± 0.2
Thermal beams		1.2 ± 0.1
Epithermal beams		1.1 ± 0.1
SAS/mp53		
γ-rays (2.0 ± 0.2^b^)	2.0 ± 0.2	2.0 ± 0.2
Carbon beams (18 keV/μm) (1.25 ± 0.15)	1.3 ± 0.1	1.3 ± 0.15
Carbon beams (50 keV/μm) (1.0 ± 0.1)	1.0 ± 0.1	1.0 ± 0.1
Thermal beams		1.2 ± 0.1
Epithermal beam		1.15 ± 0.1

^a^The ratio of the dose of radiation necessary to obtain each endpoint in the quiescent cell population to that needed to obtain each endpoint in the total tumor cell population. ^b^Mean ± standard error (n = 6).

## Discussion

PLD is the component of radiation damage that can be modified by post-irradiation conditions [24]. Under ordinary circumstances, PLD causes cell death. Changing cellular growth conditions or the microenvironment around cells influences the expression of PLD or PLDR, and thereby influences sensitivity to radiation. PLDR is favored by conditions that maintain cells without encouraging or allowing them to divide. Conditions found in solid tumors, regions of which may be far from blood vessels and low in glucose and oxygen, have a low extracellular pH, and show high concentrations of cellular waste products, may prevent cells from proliferating and thereby promote the repair of PLD. Extensive studies on PLDR suggest that DNA double-stranded breaks (dsbs) are potentially lethal lesions that can be converted into lethal damage [5]. It was reported that the conversion of potentially lethal lesions into lethal lesions might be a p53-dependent process and that PLDR was proportional to the percentage of radiation-induced DNA dsbs rejoined in 1 h in the cell lines with a normal p53 [25, 26].

Dose-rate is one of the principal factors determining the biologic consequences of a given absorbed dose. As the dose-rate is lowered and the exposure time extended, the biologic effect of a given dose is generally reduced. The dose-rate effect, which is very important in radiotherapy, results from the repair of sublethal damage (SLD) that occurs during a long radiation exposure [24]. Incidentally, SLD repair is the operational term for the increase in cell survival that is observed if a given radiation dose is split into two fractions separated by a time interval. Because continuous low dose-rate irradiation may be considered to be an infinite number of infinitely small fractions, the survival curve under these conditions also would be expected to have no shoulder and to be shallower than for a single acute exposure [24]. It was also reported that a normal functioning p53 gene is indispensable for a repair of DNA damage induced under low dose-rate irradiation [27, 28].

It is thought that decreasing the dose-rate reduces late effects in normal tissue much more than it decreases tumor control. Thus, the “therapeutic ratio” increases as the dose-rate decreases, because the therapeutic ratio is equal to the ratio of tumor control to normal tissue complications. Further, the difference between early and late effects for low dose-rate radiotherapy, as well as improving the therapeutic ratio, allows the delivery of a complete treatment in a short time, allowing the effects of tumor repopulation to be minimized. Namely, decreasing the dose-rate increases the therapeutic ratio, limited by tumor cell repopulation [24]. This is the primary rationale for low dose-rate radiotherapy. However, this rationale does not take into account the response of Q tumor cells. The current study showed that lowering the dose-rate decreases the effect on Q cells more markedly than it reduces the effect on the total cell population (Table 2). Therefore, considering the Q cell response, it follows that the therapeutic ratio does not always increase when the dose-rate is reduced.

Following γ-ray irradiation, concerning whether PLDR after HDRI or the repair during RDRI, SAS/neo showed an apparent repair phenomenon in both total and Q cell populations (Fig. 1 and Table 2). Notably, Q cells in solid tumors with wild-type p53 exhibited greater capacities of the repair than the total cell population, probably due to the intratumor conditions, that is, hypoxic, nutrition-depleted, and low pH circumstances, where Q cells came into existence [10] (Fig. 1 and Table 2). In contrast, no apparent repair was observed in total or Q cell populations within p53-mutated tumors (Fig. 1 and Table 2).

Two major pathways for the repair of potentially lethal DNA dsbs exist in mammalian cells. The non-homologous end-joining (NHEJ) pathway is imprecise, error-prone, and mutagenic, and mutant cell lines lacking key components of this pathway all exhibit impaired kinetics of DNA dsb repair and exquisite radiosensitivity [29, 30]. Homologous recombination (HR) is a more precise (error-free) repair mechanism and is more important for the repair of dsbs in late-S and G2 when a sister chromatid is available for the recombination reaction. Cell lines with defects in HR also exhibit increased radiosensitivity and decreased fidelity of repair [29, 31].

A cellular safeguard against genetic destabilization is activation of the p53 tumor suppressor protein, which commonly responds to DNA damage signals by inducing apoptosis or regulating the cell cycle including DNA damage repair [29, 32]. As also shown in our previous report [33], the net MN frequencies in SAS/neo tumor cells were lower than those in SAS/mp53 tumor cells under all conditions (P < 0.05), probably resulting from exclusion of a higher number of radiation-induced apoptotic SAS/neo cells than SAS/mp53 cells.

Loss-of-function of wild-type TP53 can result in loss of the G1/S cell-cycle checkpoint and an increase in HR [29, 32]. As p53 seems to interact with RAD51, the absence of normal p53 function is thought to enhance RAD51-mediated DNA pairing activity and HR, due to overexpression of RAD51 out of regulation by normal p53 [29, 32]. Thus, HR is thought to be a principal mechanism of DNA dsb repair in SAS/mp53 cells. The very small repair capacity of SAS/mp53 cells *in vivo* may show that the repair in solid tumors with a mutant p53 is mainly due to, if anything, the NHEJ rather than HR.

After γ-ray irradiation, the dose ratios for SAS/mp53 cells relative to SAS/neo cells showed that SAS/mp53 tumor cells within solid tumors are less radiosensitive than SAS/neo tumor cells (Table 3). This is consistent with reports that tumor cells with a mutant p53 gene were more radioresistant than those with a wild-type p53 gene [33]. Since apparent repair phenomena could be observed in solid tumors with a wild-type p53 gene, the difference in sensitivity between SAS/neo and SAS/mp53 was slightly reduced without significant differences after repair.

It is true that the RBE values can depend substantially on radiation quality like LET, radiation dose, the number of dose fractions, dose-rate, and biologic system or endpoint, including the kind of irradiated cells, tumors, and tissues [34]. However, concerning the RBE values obtained in this study, as a whole, those of the carbon-ion beams and reactor thermal and epithermal neutron beams in Q cells were significantly larger than those in total cells irrespective of p53 status of tumor cells (Table 4), reflecting the finding that Q cells showed significantly and relatively lower sensitivity than did total cells under γ-ray irradiation and under carbon-ion beams or reactor neutron beams, respectively. Nine hours was already shown to be long enough to repair the initial radiation-induced damage after γ-ray irradiation, and the capacity for PLDR was also shown to be greater in Q cells than in the total cell population [35]. In addition, the reduction in sensitivity caused by a decreasing dose-rate under γ-ray irradiation, which has been ascribed to SLD repair during infinite split-dose irradiation and PLDR during RDRI [24], was also shown to be more marked than PLDR during 9 h after HDRI following γ-ray irradiation and more clearly observed in Q cells than in total cells within solid tumors [36]. Therefore, irrespective of p53 status of tumor cells, in both cell populations, the RBE values of carbon-ion beams became larger in the following order: immediately after HDR irradiation, 9 h after HDRI, and immediately after RDRI. Furthermore, not only in the total cells, but also in Q cells, because the sensitivity to carbon-ion beams became greater as the LET value increased (i.e., as the irradiated point became deeper in the SOBP of the carbon-ion beam; Fig. 1-3), the RBE values also became larger. This means precise treatment planning for targeting tumors is essential in clinics. Otherwise, severe complications can be potentially caused in normal tissues around the target tumor. Concerning reactor neutron beams, the RBE values of the thermal neutron beams were higher than those of carbon-ion beams even with the higher LET value (50 keV/mm) and those of the epithermal beams showed significantly greater values than the others (Table 4), irrespective of p53 status of tumor cells. This means that reactor neutron beams, especially epithermal beams, have as cytotoxic, or a more cytotoxic, effect on tumor cells as a whole, including the Q cell population than do accelerated carbon-ion beams with high LET values.

Carbon-ion beams, especially with high LET values, were shown to repress the repair by leaving the interval between γ-ray irradiation and the start of the assay or decreasing γ-ray irradiation dose-rate very efficiently in the total and Q cell populations (Table 2). The difference in radiosensitivity between the total and Q cell populations was markedly reduced with the use of carbon-ion beams, especially with high LET values (Table 5). Although our previous report demonstrated that the Q cell population has a much larger hypoxic fraction than the total cell population [11], the sensitivity to carbon-ion beams of the total cell population was similar to that of the Q cells. Thus, oxygenated and hypoxic cells in solid tumors have almost the same radiosensitivity to carbon-ion beams. The difference in sensitivity between the total and Q cell populations to γ-ray irradiation was more remarkable 9 h after HDRI than immediately after HDRI because of the more apparent observed PLDR in Q cells than in the total cells [11]. However, PLDR, especially by Q cells, after γ-ray irradiation was strongly suppressed with carbon-ion beams, especially high LET beams (Table 2), resulting in a marked reduction in the difference in sensitivity between the total and Q cells (Table 5). Moreover, the reduction in sensitivity caused by a decreasing dose-rate under γ-ray irradiation, which was more marked than PLDR during the 9 h after HDRI and more clearly observed in Q cells than in total cells [36], produced greater differences in sensitivity between the total and Q cells to γ-ray irradiation than 9 h after HDRI. Nevertheless, the decrease in sensitivity by a decreasing dose-rate was also efficiently inhibited with carbon-ion beams, especially those with high LET values. Concerning reactor neutron beams, both thermal and epithermal beams also inhibited the marked reduction in sensitivity of Q cells by a decreasing dose-rate, resulting in inducing a low difference in sensitivity between the total and Q cells, similar to that with carbon-ion beams with high LET values.

Because of the lower difference in radiation sensitivity among cell cycles and a lesser capacity for PLDR and SLD repair when carbon-ion beams, especially high LET beams, were used, the RBE values in Q tumor cells 9 h after HDRI and immediately after RDRI were relatively larger than those immediately after HDRI (Table 4), irrespective of p53 status of tumor cells. These radiobiologic advantages concerning the responses of intratumor Q cell populations provided us with another reliable rationale for high LET radiotherapy. The clinical trials revealed that carbon-ion radiotherapy provided definite local control and offered a survival advantage without unacceptable morbidity in a variety of refractory tumors that include significantly more p53-mutated cells and/or hypoxic cells than normal tissues and that were hard to cure using conventional treatment modalities [18]. Additionally, only under RDRI reactor neutron beams could also cause almost similar radiosensitivity in Q cells to total cells. Taking this radiobiologic characteristic into account, reactor neutron beams, especially epithermal beams, could also be a method of solid tumor treatment. However, difficulty certainly remains in homogeneous microdistribution of ^10^B atom in target tumors and sufficient physical dose deposition at a deep site unconditionally necessary to cure and control deep-seated tumors, when the reactor neutron beams are used as a beam source for boron neutron capture therapy [17].

Solid tumors, especially human tumors, are thought to contain a high proportion of Q cells [10]. The presence of these cells is probably due, in part, to a microregional deficiency in the concentrations of oxygen, glucose, and other nutritional factors in the tumors caused by poor and heterogeneous tumor vascular supply [10]. This deficiency might promote MN formation in Q tumor cells at 0 Gy (Table 1) [10, 37]. As shown here, Q cells have lower radiosensitivity than P cells in solid tumors *in vivo*, irrespective of the p53 status of tumor cells (Table 5) [33]. This means that more Q cells survive after radiotherapy than P cells. Consequently, the control of Q cells also has a great impact on the outcome of radiotherapy. Thus, from the viewpoint of the tumor cell-killing effect including intratumor Q-cell control, a treatment modality for enhancing the Q-cell response has to be considered.

### Conclusion

In terms of tumor cell-killing effect as a whole, including Q cells, accelerated carbon-ion beams, especially with greater LET values, are very useful for suppressing the dependency on the heterogeneity within solid tumors, as well as depositing the radiation dose precisely. For tumor control, including intratumor Q-cell control, accelerated carbon-ion beams, especially with a higher LET, and reactor thermal and epithermal neutron beams were very useful for suppressing the recovery from radiation-induced damage irrespective of p53 status of tumor cells.
